# Development of a DNA damage-induced senescence model in osteoarthritic chondrocytes

**DOI:** 10.18632/aging.204881

**Published:** 2023-09-01

**Authors:** Mélina Georget, Anaïs Defois, Romain Guiho, Nina Bon, Sophie Allain, Cécile Boyer, Boris Halgand, Denis Waast, Gaël Grimandi, Alban Fouasson-Chailloux, Jérôme Guicheux, Claire Vinatier

**Affiliations:** 1 Nantes Université, Oniris, CHU Nantes, Inserm, Regenerative Medicine and Skeleton RMeS, UMR 1229, Nantes F-44000, France

**Keywords:** senescence, osteoarthritis, etoposide, IL-1β, chondrocytes

## Abstract

Senescent cells (SnCs) have been described to accumulate in osteoarthritis (OA) joint tissues in response to injury, thereby participating in OA development and progression. However, clinical therapeutic approaches targeting SnCs using senolysis, although promising in preclinical OA models, have not yet proven their efficacy in patients with knee OA. This pitfall may be due to the lack of understanding of the mechanisms underlying chondrocyte senescence. Therefore, our study aimed to generate models of chondrocyte senescence.

This study used etoposide, to induce DNA damage-related senescence or chronic exposure to IL-1β to entail inflammation-related senescence in human OA chondrocytes. Several hallmarks of cellular senescence, such as cell cycle arrest, expression of cyclin-dependent kinase inhibitors, DNA damages, and senescence-associated secretory profile were evaluated.

Chronic exposure to IL-1β induces only partial expression of senescence markers and does not allow us to conclude on its ability to induce senescence in chondrocytes. On the other hand, etoposide treatment reliably induces DNA damage-related senescence in human articular chondrocytes evidenced by loss of proliferative capacity, DNA damage accumulation, and expression of some SASP components.

Etoposide-induced senescence model may help investigate the initiation of cellular senescence in chondrocytes, and provide a useful model to develop therapeutic approaches to target senescence in OA.

## INTRODUCTION

Osteoarthritis (OA) is a debilitating disease affecting over 500 million people worldwide [1]. This disease impacts all joint tissues and is characterized by progressive cartilage degradation, synovitis, abnormal subchondral bone remodeling, and osteophyte formation [2]. These OA features lead to decreased joint function and mobility, pain, and impaired quality of life for patients [2]. Unfortunately, no disease-modifying drugs are available, and current treatments are limited to pain management and joint prosthetic replacement at the end-stage disease [3].

Among the many risk factors for OA, including obesity, female gender, history of traumatic joint injury, and genetic predisposition, aging is the primary determinant [4]. Thus, with the global aging of the population, OA is placing a growing burden on society and the economy [5].

Cellular senescence has been described as one of the major drivers of aging [6] and has been implicated in the pathogenesis of many age-related diseases [7]. Recently, cellular senescence has emerged as a new target to treat OA [8]. Indeed, senescent cells (SnCs) are described to accumulate in joint tissues in response to injury and during aging, thereby participating in its development and progression [8–10]. SnCs exhibit irreversible growth arrest accompanied by increased expression of cyclin-dependent kinase inhibitors (CDKi) such as p16^INK4a^, and p21^Cip1^, accumulation of DNA damages, and secretion of diverse bioactive molecules known as the senescence-associated secretory phenotype (SASP). This characteristic secretome includes pro-inflammatory cytokines such as interleukin (IL)-6, IL-8, and matrix-degrading enzymes such as metalloproteinases (MMPs) among others [11], and promotes tissue degradation [12] and senescence propagation [13]. Because a specific marker for cellular senescence has yet to be identified, a combination of markers should be used to identify SnCs [14]. Considering the role of cellular senescence in age-related diseases including OA, the therapeutic potential of senolytic (drugs that induced SnCs death) and senomorphic (drugs that modulate the SASP) compounds have been contemplated with growing interest [15]. In OA, while it has been shown to reduce the severity of the disease in the murine model [8, 16], the senolytic UBX0101 failed to demonstrate clinical efficacy in humans. Yet, senolytics appear of interest in other diseases as clinical trials evaluating different senolytics such as the combination of Quercetin and Dasatinib for the treatment of idiopathic pulmonary fibrosis [17] and diabetic kidney disease [18], highlighted the safety of senolytics and presented encouraging results. The search for senolytic or senomorphic compounds is therefore a booming field of research particularly in incurable and age-related diseases. However, in OA this race to identify new senescence-modifying compounds is considerably slowed by the lack of a complete understanding of the biological processes linking the accumulation of SnCs in the joint and the OA pathology. Senescent cells have been described in all joint tissues, including the synovial membrane and articular cartilage. While the fundamental processes of senescence have been extensively studied in fibroblasts, this knowledge may not be fully transferable to chondrocyte senescence, which are highly specialized post-mitotic cells. The availability of robust *in vitro* models of chondrocyte senescence could therefore help to improve our understanding of the biological mechanisms underlying chondrocyte senescence and promote the development of OA senotherapies.

*In vitro*, cellular senescence can be initiated by a range of intrinsic and extrinsic signals such as replicative stress, DNA damage, oncogenic signaling, oxidative stress, inflammation, irradiation, and/or chemotherapeutic agents [11]. Recently, IL-1β, a cytokine used *in vitro* to mimic the OA-associated inflammatory environment [19] has also been used to induce inflammation-related chondrocyte senescence [20–22]. In this IL-1β-induced chondrocyte senescence model, while senescence is a slow process to set up [23], markers of senescence were only studied over a short period (1–2 days), calling for longer-term studies to validate those previous results. Considering the variety of inductive signals potentially driving senescence, it appears reasonable to strengthen the senescence investigation by studying several models. In this context, our study aims to develop *in vitro* models of chondrocyte senescence by investigating the ability of etoposide and IL-1β treatments to produce a reliable chondrocyte senescent model.

## RESULTS

**Figure 1:**
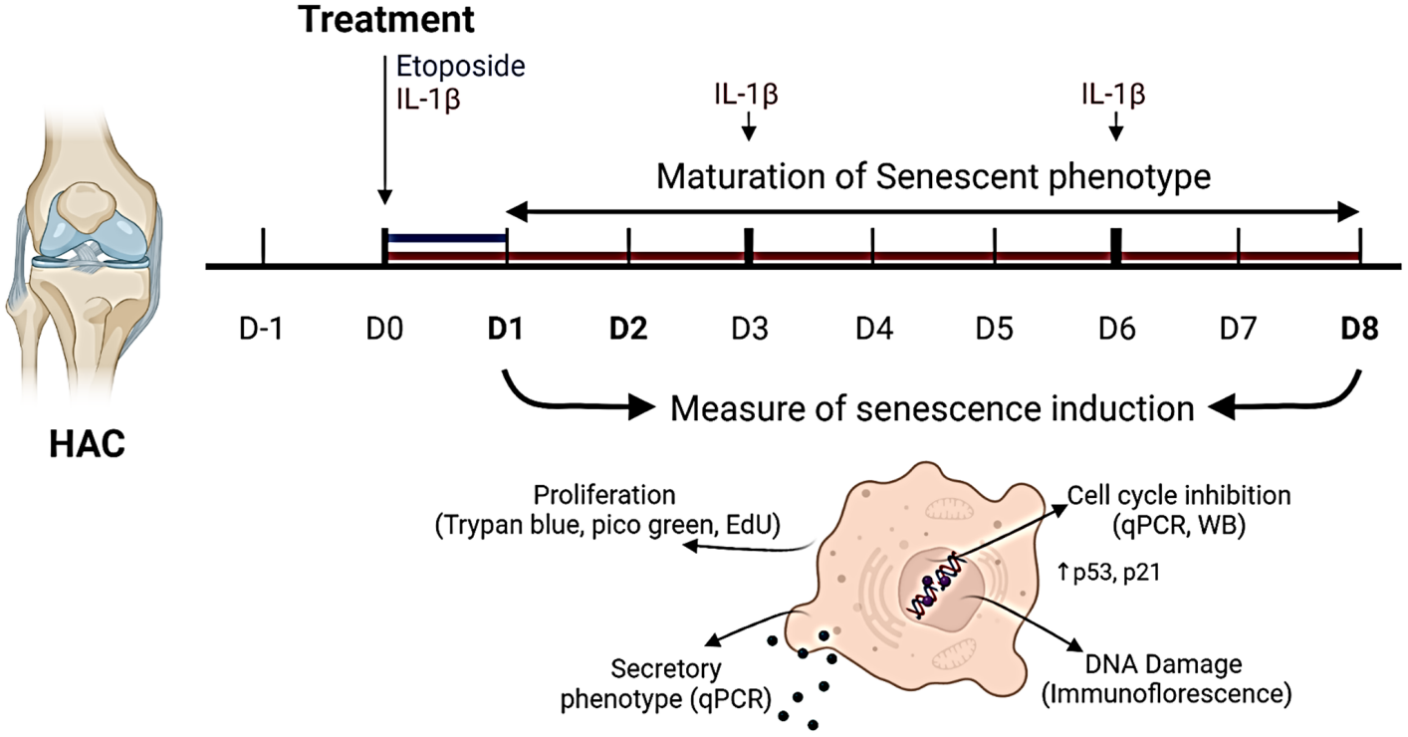
**Experimental design.** To investigate senescence in chondrocytes, primary human articular chondrocytes (HACs) were stimulated with etoposide for 24 h (blue) or with IL-1β for 8 days (red) with treatment renewal at days 3 and 6. Senescence features were assessed at days 1 and 8 in both conditions by qPCR, WB, and immunofluorescence. Figure created with https://www.biorender.com.

An overview of the experimental design is given in (Figure 1). Chondrocytes isolated from the femoral condyles of OA patients (HACs) undergoing total knee replacement surgery were treated with a concentration range of etoposide (5, 10, and 20 μM) for 24 h or chronically with recombinant human IL-1β (1 and 10 ng/mL).

### Etoposide but not IL-1β inhibits cell proliferation

**Figure 2:**
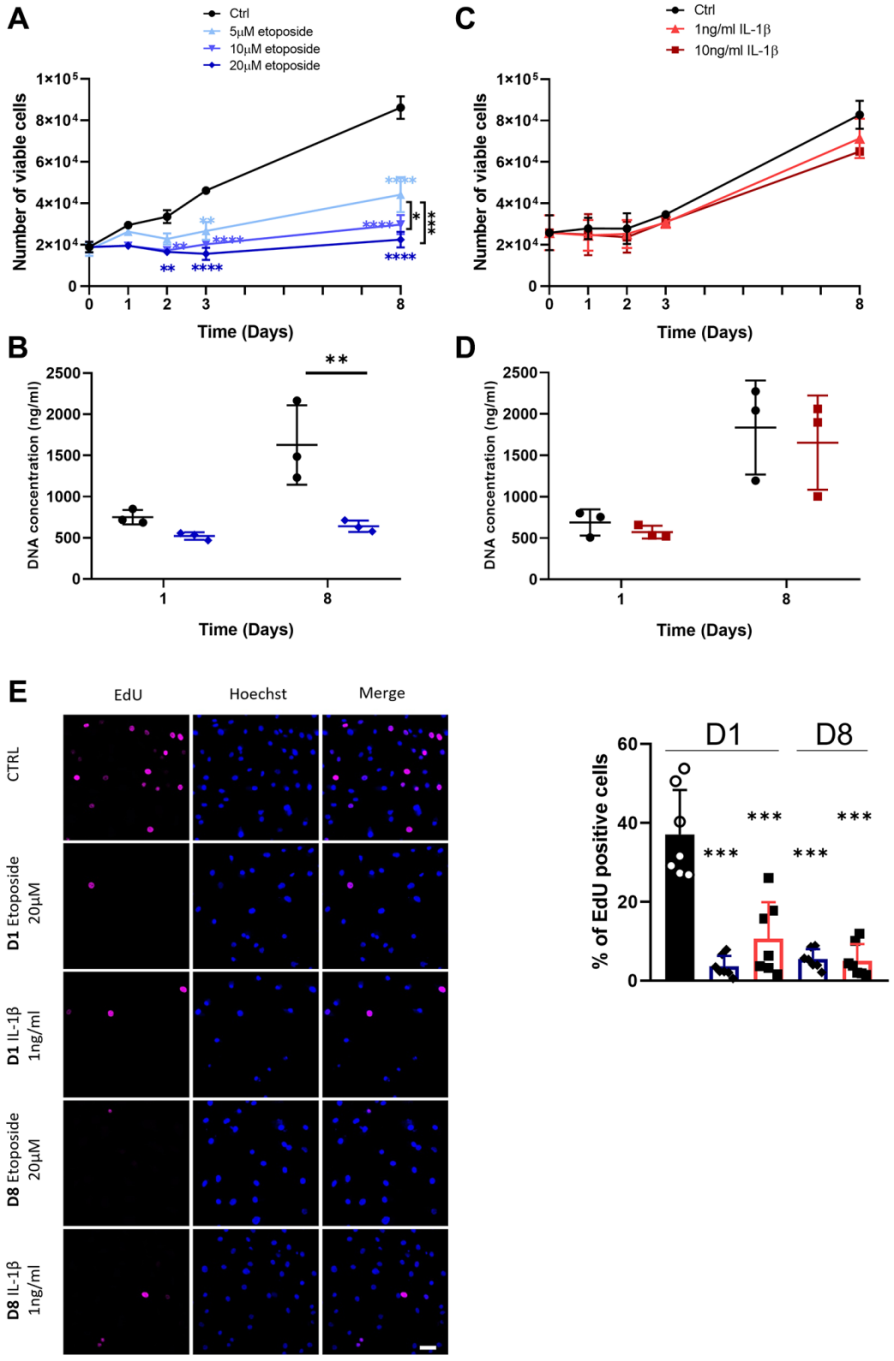
**Impact of Etoposide and IL-1β treatments on HACs proliferation.** HACs were treated with etoposide (blue) at 5, 10 or 20 μM (**A**, **B**) for 24 h and then cultured in normal media or with IL-1β (red) at 1 and 10 ng/mL (**C**, **D**) for the length of the experiment. (**A**, **C**) The number of viable cells was assessed by trypan blue exclusion dye. (**B**, **D**) DNA quantification in Etoposide and IL-1β treated HACs at day 1 and day 8. Data are shown as mean ± SD, (*n* = 3). *P*-values were calculated by the two-way ANOVA test, ^*^p ≤ 0.05; ^**^p < 0.01; ^***^p < 0.001; ^****^p < 0.0001. (**E**) EdU was used to identify proliferative cells and Hoechst staining to visualize the nucleus at day 1 and 8 (scale bar = 50 μm). The images were analyzed by quantification of positive cells for EdU normalized versus the total number of cells obtained with the Hoechst staining at each time. Data are shown as mean ± SD, (*n* ≥ 3). *P*-values were calculated by Mann-Whitney test compared to the control on day 1, ^***^p < 0.001.

To analyze at first whether etoposide and IL-1β induce a chondrocyte growth arrest, the proliferation of chondrocytes treated either with etoposide or IL-1β was evaluated. Our results indicated that etoposide-treated chondrocytes displayed a significantly reduced number of viable cells as early as day 2 compared with the control condition (Figure 2A). Moreover, only the 20 μM treatment induced stable proliferation arrest for up to 8 days and was therefore chosen for the rest of the experiments. DNA quantification assay showing significantly reduced DNA concentration at day 8 confirmed these results (Figure 2B). Similar results were obtained with the chondrocyte line TC28a2 treated with 20 μM etoposide (Supplementary Figure 1A).

On the contrary, IL-1β treatment (Figure 2C), regardless of the dose used (1 or 10 ng/mL) failed to halt cell proliferation, as indicated by similar cell viability and DNA concentration between control and IL-1β treated HACs (Figure 2C, 2D). In order to clarify the viable cell number, decreased in the treated conditions, overnight EdU incorporation was measured. Quantification of EdU-positive cells by immunofluorescence showed that EdU incorporation was significantly reduced in etoposide and IL-1β conditions at day 1 and day 8 compared with the control at day 1, indicating a reduced cell proliferation (Figure 2E, Supplementary Figure 2A). The apparent contradiction between the high number of viable IL-1β-treated cells at D8 and the proliferation arrest observed with EdU at the same time may result from contact inhibition when chondrocytes reach maximum confluence.

These data suggest that treatment with 20 μM etoposide for 24 h sustainably impaired cell-cycle progression. Conversely, chronic IL-1β treatment only transiently impacts cell proliferation.

### Etoposide treatment induces the expression of CDKi

**Figure 3:**
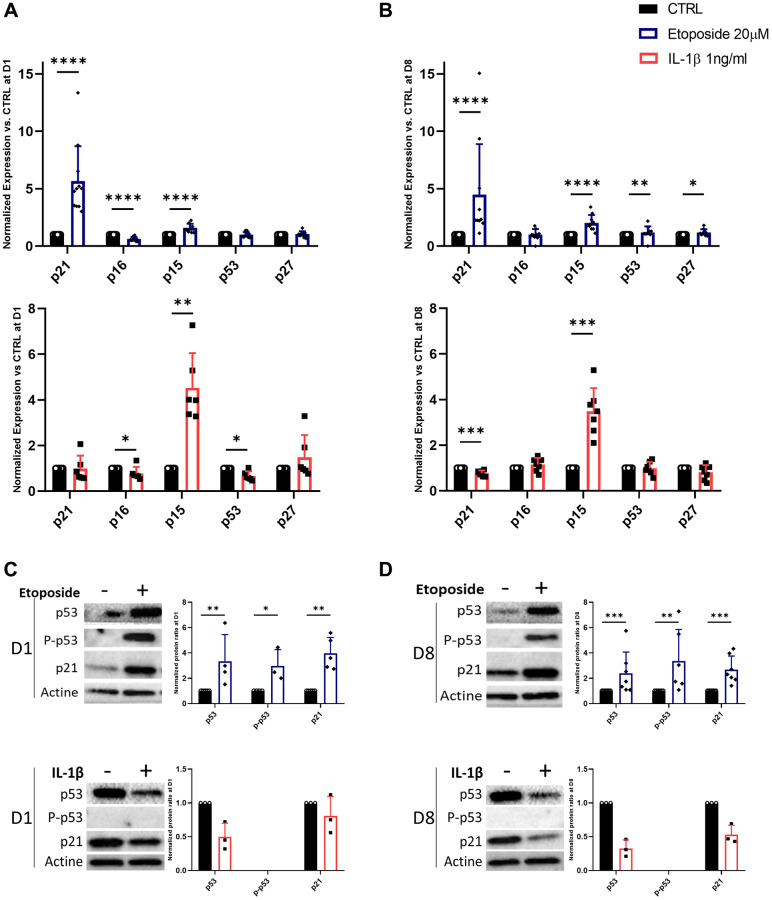
**Expression of cyclin-dependent kinase inhibitors in Etoposide, and IL-1β treated HACs.** HACs were treated with etoposide (blue) at 20 μM for 24 h or IL-1β (red) at 1 ng/mL for the length of the experiment and the expression of cyclin-dependent kinase inhibitors evaluated by RT-qPCR (**A**, **B**) and WB (**C**, **D**) at day 1 (**A**, **C**) and day 8 (**B**, **D**). Data are shown as mean ± SD, (*n* ≥ 3). *P*-values were calculated by Mann-Whitney test, *p ≤ 0.05; ^**^p < 0.01; ^***^p < 0.001; ^****^p < 0.0001.

Since the stable growth arrest of SnCs is linked to the engagement of CDKi, we investigated whether etoposide or IL-1β can induce the expression of several CDKi at the transcriptional and protein level (Figure 3A–3D). HACs treated with etoposide displayed as early as day 1, a significant increase in the expression of two CDKi, p21^CIP1^ (*CDKN1A*) (5.65-fold change) and p15^INK4b^ (*CDKN2B*) (1.59-fold change) compared to non-treated cells (Figure 3A). Their expressions remained significantly elevated throughout the experiment until day 8 (Figure 3B). A significant increase in p53 (1.18-fold change) and p27 (CDKN1B) (1.19-fold change) was also observed on day 8. Surprisingly, p16^INK4a^ (*CDKN2A*) expression was significantly diminished (0.62-fold change) following etoposide treatment on day 1 (Figure 3A), but remained equivalent to the control condition on day 8 (Figure 3B). Since p16^INK4a^ expression could be a late senescence marker, we evaluate its expression (RNA and protein level by immunofluorescence) on day 15 and still observed comparable expression to the control condition (data not shown). In line with the transcriptomic results, the protein level of p21 was also significantly increased at both time points and the p53 phosphorylated activated and total forms were increased (2.98-fold and 3.35-fold respectively) after 24 h of etoposide treatment and stayed up-regulated until the end of the experiment (3.37-fold and 2.39-fold respectively) (Figure 3C, 3D). These results showed that the p53-p21^CIP1^ axis was involved. Similarly, in the TC28a2 chondrocytes line, 20 μM etoposide treatment enhanced p21^CIP1^ expression on days 1 and 8 (Supplementary Figure 1B).

Surprisingly, in IL-1β-treated HACs despite the lack of impact of IL-1β treatment on cell proliferation, p15^INK4b^ expression is significantly up-regulated from day 1 (4.53-fold) and remained so until day 8 (3.48-fold) (Figure 3A, 3B). However, on day 8 p21^CIP1^ expression is down-regulated 0.77-fold (Figure 3B). At day 1, p16^INK4a^ and p53 are also down-regulated (0.78 and 0.67-fold changes respectively) (Figure 3A). At the protein level (Figure 3C, 3D), the phosphorylated form of p53 was undetectable, and its total form was reduced in treated HACs versus HACs control at each time point. In line with the decrease in p21^CIP1^ gene expression on day 8, its protein level was reduced.

### Etoposide promotes cellular senescence by inducing DNA damage accumulation

**Figure 4:**
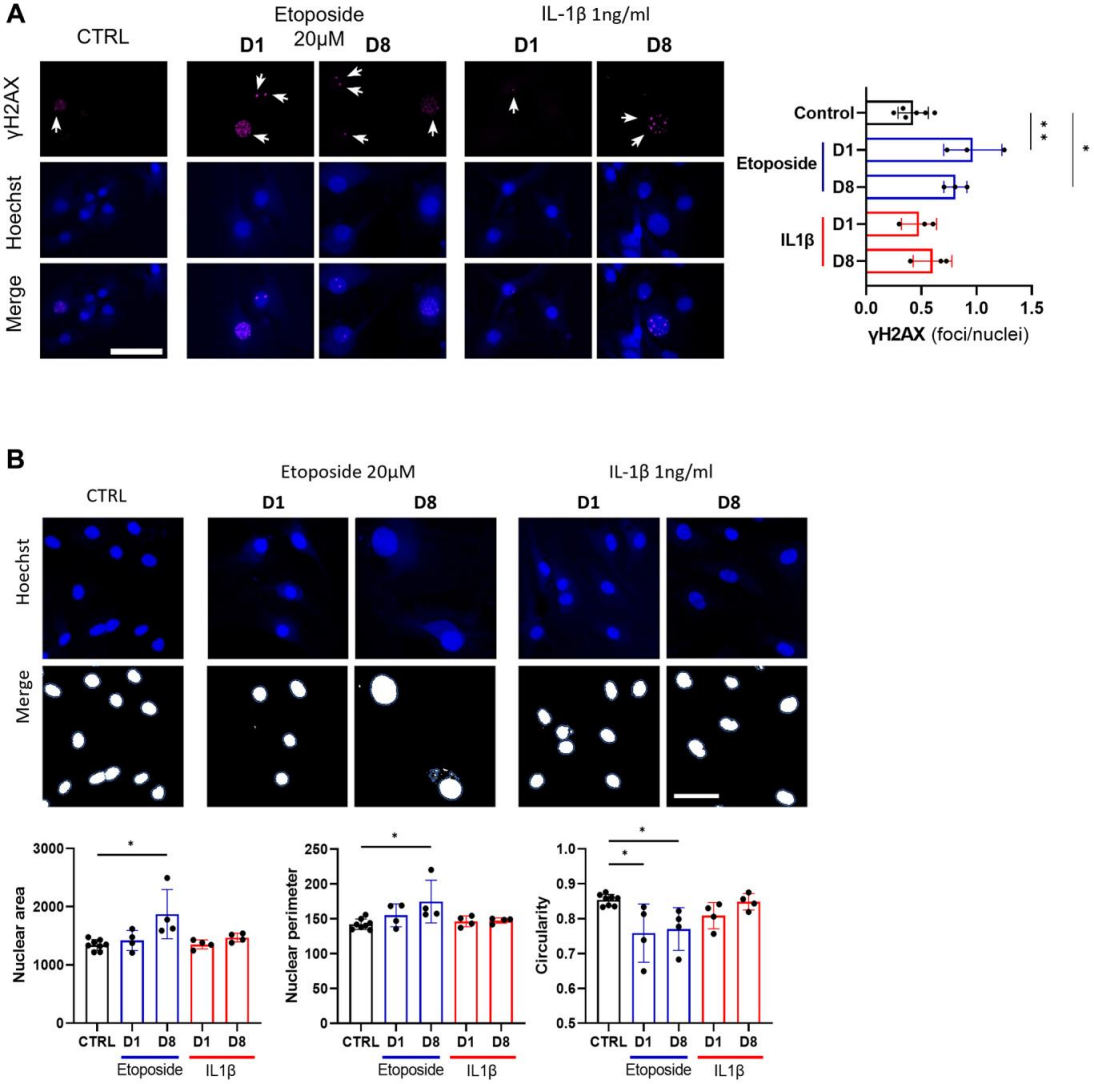
**DNA damage assessment and senescence-associated nuclear features measure in Etoposide, and IL-1β treated HACs.** HACs were treated with Etoposide 20 μM (blue) for 24 h or chronically with 1 ng/mL IL-1β (red) for the length of the experiment. (**A**) γH2AX immunofluorescence was used to identify DNA damage-associated foci and Hoechst staining to visualize the nucleus at day 1 and day 8. Quantification of the average number of foci per nuclei is shown. (**B**) Nucleus surface, perimeter and circularity was analyzed using CellProfiler software on the Hoechst channel. Scale bars = 50 μm. Data are shown as mean ± SD, (*n* = 3). *P*-values were calculated by Kruskal-Wallis test, ^*^p ≤ 0.05; ^**^p < 0.01.

Since etoposide is a topoisomerase II inhibitor known to induce DNA damages, we analyzed the number of γH2AX foci in HACs, the accumulation of these foci being a cellular marker of DNA damage (Figure 4A). At day 1, numerous etoposide-treated cells started to exhibit γH2AX foci, resulting in a global increase of average foci per nuclei ratio, this higher ratio was sustained up to day 8 in this condition. On the contrary, chronic IL-1β treatment, even with the higher dose of 10 ng/mL (Supplementary Figure 2B), failed to impact the γH2AX foci per nuclei ratio in HACs, indicating the absence of DNA damage in this condition. Recently, SnCs have been characterized by hypertrophy and enlarged and less circular nuclei [24], therefore we also measured nuclei sizes and circularity across our conditions (Figure 4B). On day 1, no difference in nucleus size (i.e., area and perimeter) was observed between the conditions whereas the nuclear shape of etoposide-treated HACs was already less circular than the control nuclei. At day 8, etoposide-treated HACs displayed significantly enlarged nuclei (1.6-fold for the area, 1.2-fold for the perimeter) compared to control cells. The nuclei circularity of etoposide-treated HACs at day 8 remained lower. Together, these data reinforce that a short 20 μM etoposide treatment for 24 h is able to induce a cellular senescent state in HACs.

### Inflammation and SASP markers in HACs

**Figure 5:**
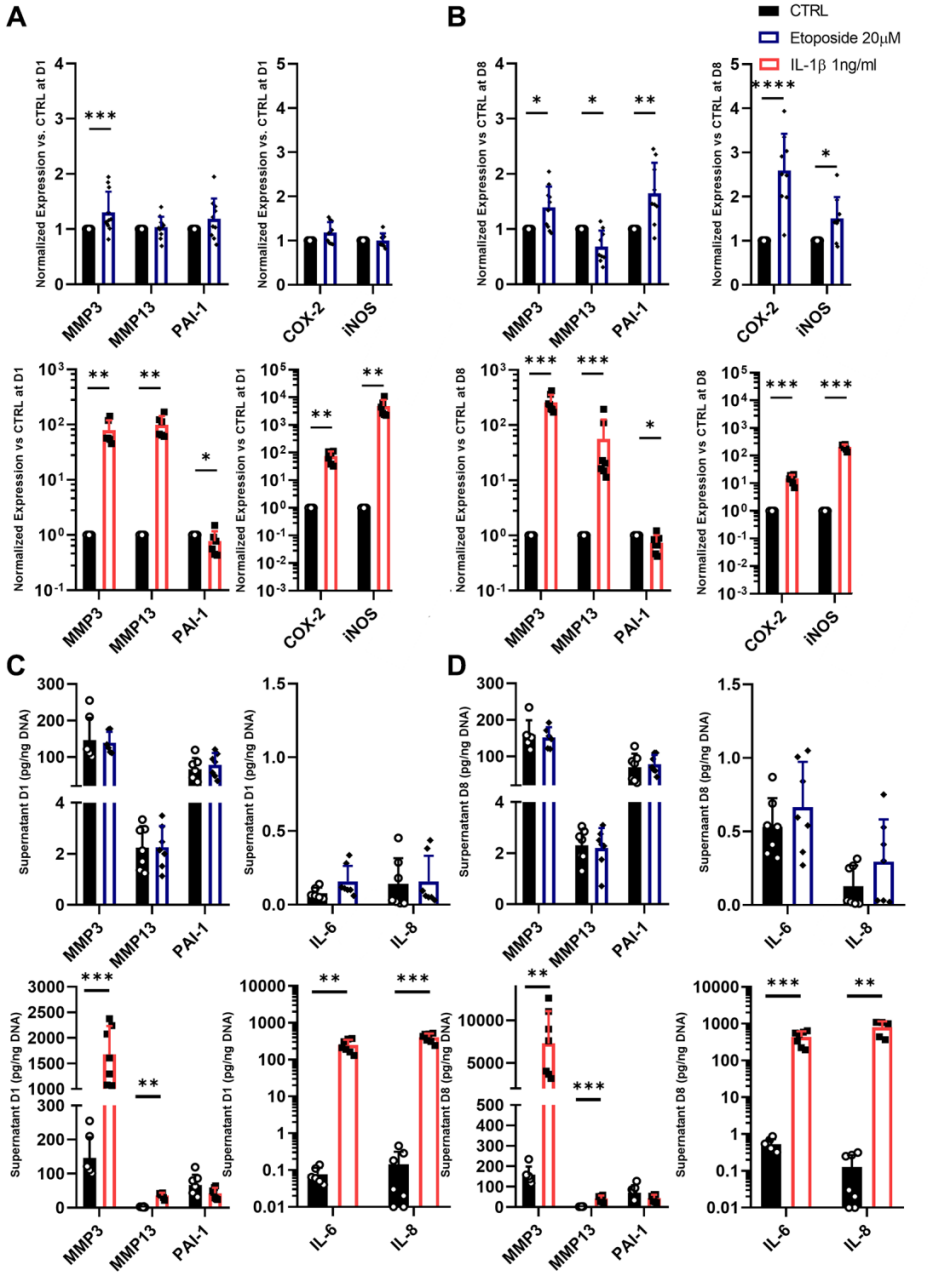
**SASP markers in Etoposide and IL-1β treated HACs.** (**A**–**D**) HACs were treated with Etoposide (blue) at 20 μM for 24 h or IL-1β (red) at 1 ng/mL for the length of the experiment. The expressions of SASP markers were evaluated by RT-qPCR at day 1 (**A**) and day 8 (**B**), and SASP components were quantified by Luminex assay in the culture medium at day 1 (**C**) and 8 (**D**). Data are shown as mean ± SD, (*n* ≥ 3). *P*-values were calculated by Mann-Whitney test, ^*^p ≤ 0.05; ^**^p < 0.01; ^***^p < 0.001; ^****^p < 0.0001.

As the secretion of SASP is one of the four hallmarks of SnCs defined by the International Cell Senescence Association (ICSA), we next assessed the gene expression and secretion of several SASP markers following our treatments with etoposide or IL-1β. Among the various SASP components, we investigated proinflammatory cytokines such as IL-6 and IL-8, the matrix metalloproteinase-3 (MMP3), MMP13, and the plasminogen activator inhibitor-1 (PAI-1), three enzymes implicated in cartilage degradation and fibrosis, and the cyclooxygenase-2 (COX-2) and the inducible nitric oxide synthase (iNOS) two enzymes respectively involved in the synthesis and production of prostaglandins and nitric oxide; two factors known to promote and supports inflammation (Figure 5A, 5B).

Etoposide treatment at day 1 induced significant but weak up-regulation in the mRNA expression level of MMP3 (1.30-fold change) and a slight tendency to increase PAI-1 (1.19-fold change) mRNA expression (Figure 5A). At day 8, MMP3, PAI-1, COX-2, and iNOS were significantly increased by etoposide treatment (Figure 5B). On the contrary, MMP13 expression was either not modified on day 1, or significantly decreased on day 8 following etoposide treatment. Similar results were also obtained in the TC28a2 cell line (Supplementary Figure 1C). However, despite the increased expression at the transcriptomic level, none of these observations were confirmed at the protein level. Indeed, no increased levels of MMP3 and PAI-1 were found in the supernatant of etoposide-treated chondrocytes (Figure 5C, 5D). Concerning the secretion of the two pro-inflammatory cytokines, IL-6 and IL-8, etoposide treatment only tends to increase the IL-6 secretion at both day 1 and day 8.

At day 1, IL-1β, as expected, significantly up-regulated the mRNA level of MMP3 (79.04-fold change), MMP13 (99.49-fold change), COX-2 (74.97-fold change), and iNOS (4810.8-fold change) (Figure 5A). Only PAI-1 gene expression was decreased (0.77-fold change). Overall, at day 8, the gene expression of the previous markers remained similar to day 1 (Figure 5B). IL-1β enhanced the secretion of all markers including IL-6 and IL-8 as early as day 1 except for PAI-1 which tends to be reduced (Figure 5C, 5D). Interestingly, PAI-1 showed opposite profiles between the etoposide and IL-1β treatments, evidencing that despite sharing several markers, senescence and inflammation had different gene expression profiles for secretory molecules with variations in their intensity and kinetics.

## DISCUSSION

With the emergence of senescence-targeted therapeutic strategies for OA, the mechanisms involved in chondrocyte senescence are being increasingly studied. However, there is currently a lack of human chondrocyte senescence models to study or screen potential treatments targeting chondrocyte senescence. In this study, we investigated two types of senescence inducers, etoposide, and IL-1β on chondrocytes to develop a robust model of chondrocyte senescence. Since OA chondrocytes are hypo-replicative cells and display a high level of DNA damage which could contribute to chondrocyte senescence [25, 26], we aimed to generate a model of DNA damage-induced senescence in chondrocytes. To this end, etoposide, a topoisomerase II inhibitor known to induce senescence through the induction of double-strand breaks and the activation of the DNA damage response in other cell types [27, 28] was investigated as a chondrocyte senescence inducer. Furthermore, given the interconnection of chronic low-grade inflammation and senescence [29], we also investigated whether chronic exposure to IL-1β could induce inflammation-related senescence in chondrocytes.

This study demonstrates that etoposide treatment of HACs reliably induces a DNA damage-related senescence. On the contrary, chronic treatment of IL-1β induces only partial expression of senescence markers and does not allow us to conclude on its ability to induce chondrocyte senescence. Indeed, in the absence of a universal and specific senescence marker, it may be challenging to differentiate senescence from other biological processes, especially between SASP and inflammation, whose markers overlap. In this regard, it is essential to characterize multiple biomarkers [11] to fully identify SnCs.

Our results indicate that, in our conditions, IL-1β does not trigger a permanent growth arrest. Since cell cycle arrest is one of the four interdependent hallmarks of senescence with SASP, macromolecular damage, and deregulated metabolism, this result questions the ability of IL-1β to induce cellular senescence, contrary to what has been assumed in various studies using IL-1β treatment [20–22]. Conversely, our etoposide model displays inhibition of proliferation even 8 days after the end of the treatment. Moreover, differences in the CDki engaged following etoposide and IL-1β treatments are observed. Indeed, contrary to IL-1β, etoposide activates the p53/p21^CIP1^ pathway in chondrocytes, consistent with the DNA damage-related senescence induced by etoposide and reported in other cell types [28, 30].

Unexpectedly, the expression of the cyclin-dependent kinase 4 inhibitor B, also known as p15^INK4b^ and encoded by *CDKN2B* is up-regulated following both etoposide and IL-1β treatments. p15^INK4b^ is also a member of the CDKi family, which forms a complex with CDK4 or CDK6, and prevents the activation of the CDK by cyclin D [31]. However, while p15^INK4b^ is well-known for its role in cell cycle progression, it does not seem to impact cell growth in our experiments since IL-1β–treated cells exhibit a similar proliferation profile to the control condition. p15^INK4b^ is downstream Raf-Mek-Erk and the PI3K/AKT/FOXO3 pathways [11] and wields other functions such as regulation of smooth muscle cells apoptosis [32, 33] or regulation of hematopoietic cell fate [34]. In our experiments, its expression could be linked to apoptosis resistance due to cellular stress induced by both treatments, but this hypothesis should be investigated in future works.

In our etoposide-induced senescent chondrocytes model, some senescent features take time to occur and are only visible 8 days after the transient etoposide stimulation. Indeed, if etoposide induces both accumulations of γH2AX foci, a reduced Edu incorporation from day 1 after treatment, nuclei enlargement can only be detected from day 8. The accumulation of γH2AX foci is a typical feature of the persistent double-strand break DNA damage in cellular senescence.

Interestingly, in our IL-1β condition, these senescent features are not found even after 8 days. Given that an increase in the number of γH2AX positive cells in response to IL-1β has been reported [35, 36] this lack of increased γH2AX accumulation may be surprising. However, even with a higher dose of IL-1β (10 ng/mL) similar to the dose used in the literature, we did not observe an increase in γH2AX-positive cells. Whether that could be related to differences in the cell model used remains to be further investigated.

Thirdly, we examined the gene expression of several SASP components. Among the described SASP markers, we selected seven SASP factors-related genes or proteins, IL-6, IL-8, MMP3, MMP13, COX-2, iNOS, and PAI-1. As expected, IL-1β induced the expression of IL-6, IL-8, MMP3, MMP13, COX-2, and iNOS, which have historically been associated with inflammation in chondrocytes [37] and not solely with SASP. Our etoposide-induced senescent model also displayed elevated expression of MMP3, COX-2, and iNOS but of a lower magnitude than the inflammation response triggered by IL-1β. Moreover, etoposide only tends to increase the secretion of IL-6, whereas IL-1β, as expected, strongly increases both IL-6 and IL-8 secretion. The lower level of expression of inflammation markers would be more consistent with the inflammaging concept [38], where chronic, low-grade inflammation promotes aging and is a major risk factor for age-related conditions such as Alzheimer's disease, atherosclerosis, macular degeneration, and OA [39, 40], and also as promoting senescence.

Interestingly, results from our study showed that senescent chondrocytes tend not to over-express MMP13. This is in line with other research teams' results [41, 42], where senescence was induced with irradiation alone or combined with mitogenic stimulation using TGF-β and bFGF on cartilage explants. The authors speculate that this may be because each senescent marker highlights a different subset of SnCs. But intriguingly, this observation remains consistent across all our studies, despite the use of different stimuli to induce senescence.

It is also worth noting that conversely to the IL-1β inflammation model where the expression of PAI-1 (SERPINE1) is decreased in line with previous observations in human articular cartilage [43], PAI-1 expression is upregulated in our etoposide-induce senescence model. PAI-1 is part of the serine-protease inhibitors and the principal physiological inhibitor of the plasminogen activators, urokinase (uPA), and tissue plasminogen activator (tPA) [44]. PAI-1 activity is known to increase with age, leading to reduced fibrinolytic activity. This reduced fibrinolytic activity participates in matrix tissue homeostasis disruption, promoting a profibrotic environment, and loss of tissue elasticity [44]. The link between PAI-1 and senescence is well established in several models of senescence [45–47] and PAI-1 is considered a key marker of cellular senescence that contributes to the progression of various age-related diseases in humans such as emphysema, arterial thrombosis, arteriosclerosis, and hypertension [48, 49]. PAI-1 has been identified as a fundamental component of the SASP, using a range of senescence induction stimuli such as replication, oxidative stress, chemotherapeutic agents, and irradiation in several human cell types [50]. The expression of PAI-1 has also been found to be significantly increased in human synovial tissues from OA patients [51, 52]. We, therefore, propose that PAI-1 could serve as a marker of chondrocyte senescent SASP. As the SASP likely contributes to promote senescence in neighbouring cells it could be relevant to develop inhibitors to dampen PAI-1 activity to slow cellular senescence spreading as well as prevent aging-related diseases including OA.

To complete our investigation on the reliability of the etoposide-induced DNA damage-related senescence in chondrocytes, we have demonstrated that etoposide is also able to induce a senescence-like state in the immortalized human chondrocytes cell line TC28a2. In this cell line, the expression of the SV40 large T antigen forces TC28a2 cells to proliferate by inhibiting both p53 and Rb-dependent regulation of the cell cycle. However, a senescent-like phenotype can be observed following cytostatic treatment by etoposide that needs a more detailed characterization. The use of the TC28a2 cell line offers the advantage of circumventing several limitations associated with the utilization of primary articular chondrocytes such as sample harvesting and preparation, the limited number of chondrocytes extracted per sample, and the inter-individual variability. Even if this model is very artificial and may be limited to specific aspects of the complex senescent phenotype (e.g., SASP, apoptotic resistance mechanisms, metabolic alterations), the TC28a2 may enable the use of high throughput screening techniques to identify new senolytic and senomorphic compounds to target SnCs as a potential treatment for OA.

In conclusion, etoposide treatment was able to induce senescence and senescence-like state in two types of human articular chondrocytes, HACs, and the TC28a2 cell line. In this model, senescence is evidenced by loss of proliferative capacity, DNA damage accumulation, and SASP production. This etoposide-induced senescence model may help investigate the initiation of cellular senescence in chondrocytes since a better comprehension of senescence induction and phenotype will help to develop therapeutic approaches to target senescence in OA.

## METHODS

### Isolation and amplification of primary human articular chondrocytes (HACs)

Primary human OA articular chondrocytes (HACs) were isolated from the femoral condyles of OA patients undergoing total knee arthroplasty. All samples were harvested from patients after their informed consent according to the Declaration of Helsinki. This study was carried out following the recommendation of the Committee for Person Protection of Pays de La Loire and approved by the French Ministry of Higher Education and Research (registration number: DC-2017-2987).

Upon reception, human articular cartilage was cut into small slices and digested at 37°C with 0.2% type II collagenase for 30 min. Finally, slices were digested overnight at 37°C in 0.03% collagenase in DMEM (DMEM-Glutamax, 61965-026, Gibco) containing 10% fetal calf serum (FCS, S1810-500, Dominique Dutscher) and 1% penicillin/streptomycin (PS, 1000 U/mL, 15140122, Thermo Fisher Scientific) (complete medium). Cell suspension was then filtered through a cell strainer with 70 μm pores and cells plated at 6.0 × 10^4^ cells/cm² (passage 0 (P0)) density. HACs were cultured in a complete medium. The cells were maintained at 37°C in a humidified atmosphere of 5% CO_2_ and the culture medium was changed every 2–3 days. For all the experiments, the HACs were used at P1.

### Culture of TC28a2 chondrocytes

The TC28a2 chondrocytes (SCC042, Sigma Aldrich) were seeded at a density of 15 000 cells/cm² in a complete medium renewed every 2–3 days and cultured in a humidified atmosphere with 5% CO_2_. Cells were subcultured when they reached 80–90% confluence. For the experiments, TC28a2 chondrocytes were used between passages 5 and 9.

### Chondrocytes treatments

The experimental layout is provided in Figure. 1. At Day-1 (D-1) chondrocytes were plated at a density of 15 000 cells/cm² in a complete medium in a humidified atmosphere with 5% CO_2_ and allowed to adhere for 24 h. At D0, cells were treated either with 1 or 10 ng/mL of IL-1β (IL038, Millipore) for up to 8 days (red), and medium with IL-1β was replaced every 3 days, or with etoposide (E1383, Sigma-Aldrich), at 5, 10 or 20 μM for 24 h (blue) and the medium replaced by fresh complete medium without etoposide for the rest of the experiment, as previously described [23]. Subsequent analyses were performed 1- and 8-days post-treatment as illustrated in Figure 1.

### Cell proliferation assay and PicoGreen analysis

At each indicated time, chondrocytes were trypsinized and the number of living cells was counted using Trypan Blue exclusion dye. Total DNA content was quantified with Pico-green (Quant-iT™ PicoGreen dsDNA Reagent, Invitrogen™, P11496) according to the manufacturer’s instructions.

### RNA extraction and quantitative polymerase chain reaction (qPCR)

RNA was extracted using a NucleoSpin^®^ RNA XS kit (74092, Macherey-Nagel, Hoerdt, France) according to the manufacturer’s instructions. RNA yield was measured using NanoDrop™ 1000 Spectrophotometers (Thermo Scientific™). Reverse transcription was performed using the Verso cDNA Synthesis Kit (AB1453B, Thermo Scientific™). Real-time polymerase chain reaction (PCR) was performed using specific primers (Table 1) with SYBR™ Select Master Mix (4472908, Applied Biosystems™) on the CFX96 Touch Real-time PCR Detection System (Bio-Rad). GUS-B and PPIA were used as reference genes and results were expressed as relative expression levels using the Pfaffl method [53].

**Table 1:** List of PCR primers.

**Gene**	**Forward**	**Reverse**
**CDKN1A - p21^CIP1^**	5′-GCAGACCAGCATGACAGATTTC-3′	5′-GCGGATTAGGGCTTCCTCTT-3′
**CDKN2A - p16^INK4a^**	5′-AAGGTCCCTCAGACATCCCC-3′	5′-CCCTGTAGGACCTTCGGTGAC-3′
**CDKN2B - p15^INK4b^**	5′-GCGGGGACTAGTGGAGAAG-3′	5′-CTCCCGAAACGGTTGACTC-3′
**p53**	5′-CCTCAGCATCTTATCCGAGTGG-3′	5′-TGGATGGTGGTACAGTCAGAGC-3′
**CDKN1B - p27**	5′-ATAAGGAAGCGACCTGCAACCG-3′	5′-TTCTTGGGCGTCTGCTCCACAG-3′
**MMP3**	5′-CACTCACAGACCTGACTCGGTT-3′	5′-AGCAGGATCACAGTTGGCTGG-3′ -3′
**MMP13**	5′-CCAGTCTCCGAGGAGAAACA-3′	5′-AAAAACAGCTCCGCATCAAC-3′
**PAI-1**	5′-CTCATCAGCCACTGGAAAGGCA-3′	5′-GACTCGTGAAGTCAGCCTGAAAC-3′
**COX-2**	5′-CTTCACGCATCAGTTTTTCAAG-3′	5′-TCACCGTAAATATGATTTAAGTCCAC-3′
**iNOS**	5′-GCTCAAATCTCGGCAGAATC-3′	5′-GCCATCCTCACAGGAGAGTT-3′
**Gus-b**	5′-CGCCCTGCCTATCTGTATTC-3′	5′-TCCCCACAGGGAGTGTGTAG-3′
**PPIA**	5′-ATGCTGGACCCAACACAAAT-3′	5′-TCTTTCACTTTGCCAAACACC-3′

### Western blot

Whole-cell lysates were collected and lysed in ice-cold RIPA buffer (50 mM Tris-HCl (pH 8), 150 mM NaCl, 1 mM Nonidet P-40, 0.5 mM Sodium deoxycholate, 20 mM EDTA) supplemented with protease and phosphatase inhibitor mixture (Sigma Aldrich, P8340 and P044). Protein concentration was measured with the Pierce™ BCA Protein Assay Kit (Thermo Scientific™, 23225). 20 μg of proteins were separated by sodium dodecyl sulfate-polyacrylamide gel electrophoresis (SDS PAGE) on a 4–15% polyacrylamide Criterion™ TGX Stain-Free™ gel (BioRad) and transferred into polyvinylidene difluoride (PVDF) membranes (Trans-Blot Turbo Midi 0.2 μm PVDF Transfer Packs BioRad) using a Trans-Blot Turbo System (BioRad). Membranes were blocked in 5% non-fat dry milk or p53, P-p53, and actin or 3% BSA for p21 in tris-buffered saline 1% tween (TBS-T). The membranes were immunoblotted with the following antibodies: anti-p53 (Cell Signaling Technology, sc126, 1/1000), anti-P-p53 (Cell Signaling Technology, 9284, 1/1000), anti-p21 (Abcam, ab109520, 1/5000); and anti-Actin (Sigma Aldrich, A2228, 1/5000) overnight at 4°C in TBS-T 5% non-fat dry milk.

Blots were incubated with HRP conjugated secondary antibodies (Cell Signaling Technology, 7074S, 1/2 000 or 7076 CST, 1/80 000 in TBS-T 5% non-fat dry milk), and signals detected with SuperSignal™ West Femto (Thermo Scientific™, 34094) or SuperSignal™ West Dura (Thermo Scientific™, 34075). The images of the blots were analyzed with Image Lab software (Bio-Rad Laboratories, version 6.1.0) and normalized versus actin.

### Supernatant analysis

Culture supernatants were collected on days 1 and 8 and stored at −80°C until analysis. In parallel, the DNA content of each condition was quantified with Pico-green analysis as previously described. The concentrations of IL-6, IL-8, MMP3, MM13, and PAI-1 in the supernatant were measured simultaneously using a multiplex cytokine assay. The human Luminex discovery assay (LXSAHM, Bio-Techne Ltd.) was used according to the manufacturers, and measurements were performed using Bio-PlexTM200 System (Bio-Rad Laboratories) plate reader. The concentration obtained for each cytokine and enzyme was normalized versus the DNA content of the matching condition.

### Immunofluorescence assays

For EdU (5-ethynyl-2’-deoxyuridine) staining, HACs were seeded on glass coverslips placed in MW24. At the end of days 1 and 7, Edu (Click-iT™ EdU Cell Proliferation Kit for Imaging, Alexa Fluor™ 488 dye, Invitrogen™) was added to culture media at a final concentration of 10 μM and incubated overnight at 37°C. HACs were fixed with 4% paraformaldehyde (PFA) at RT for 15 min and rinsed 3 times in phosphate-buffered saline (PBS) 1X and stored in PBS 1X, 1% PFA at 4°C until the end of the experiment. The cells were permeabilized with 0.5% Triton X-100 in PBS for 20 min and rinsed twice in PBS 1X, 3% BSA. Click-iT^®^ reaction cocktail was prepared according to the manufacturer’s instructions and applied for 30 min at room temperature (RT), protected from light. The cells were then washed once with PBS 1X, 3% BSA. Subsequently, for phosphorylated histone H2AX on serine 139 (γH2AX) staining, cells were incubated with 5% normal goat serum and 0.3% triton in PBS for 1 h at RT. The blockage solution was then removed and HACs were incubated with γH2AX antibody (Cell Signalling Technology, 9718, 1/400) at 4°C overnight. The next day, cells were washed 3 times in PBS and incubated with Alexa Fluor 647 goat anti-rabbit IgG (Thermo Fisher Scientific, A21244, 1/500) for 1 h at RT and with Hoechst 2 ug/mL for 20 min. Coverslips were mounted with ProLong™ Gold Antifade Mountant (Invitrogen™, P36930). Images were acquired with a confocal microscope (Nikon A1R-s Confocal Microscope) and quantifications were performed using the Image J software, to measure number of γH2AX foci and EdU positivity; at least 100 cells per condition were analyzed. Nuclear shape profiling was determined on Hoechst channel using CellProfiler software v4.2.5 [54].

### Statistical analyses

Statistical analyses and plotting were performed using GraphPad 8 Prism Software. For all experiments, outliers were identified using the Grubbs test with α = 0.05 and were excluded from subsequent analysis. The data were analyzed by Two-way ANOVA with Tukey’s multiple comparisons or Mann-Whitney and Kruskal-Wallis tests for non-parametric values. *P* values equal to or less than 0.05 were considered significant, ^*^p ≤ 0.05; ^**^p < 0.01; ^***^p < 0.001; ^****^p < 0.0001.

## Supplementary Materials

Supplementary Figures
