# Supplementary material for: Development of a DNA damage-induced senescence model in osteoarthritic chondrocytes
Source: Aging (Albany NY). 2023 Sep 1;15(17):8576–93. doi: 10.18632/aging.204881 (PMC10522398; doi:10.18632/aging.204881)
Supplement: Supplementary Figures [file aging-15-204881-s001.pdf]

SUPPLEMENTARY FIGURES

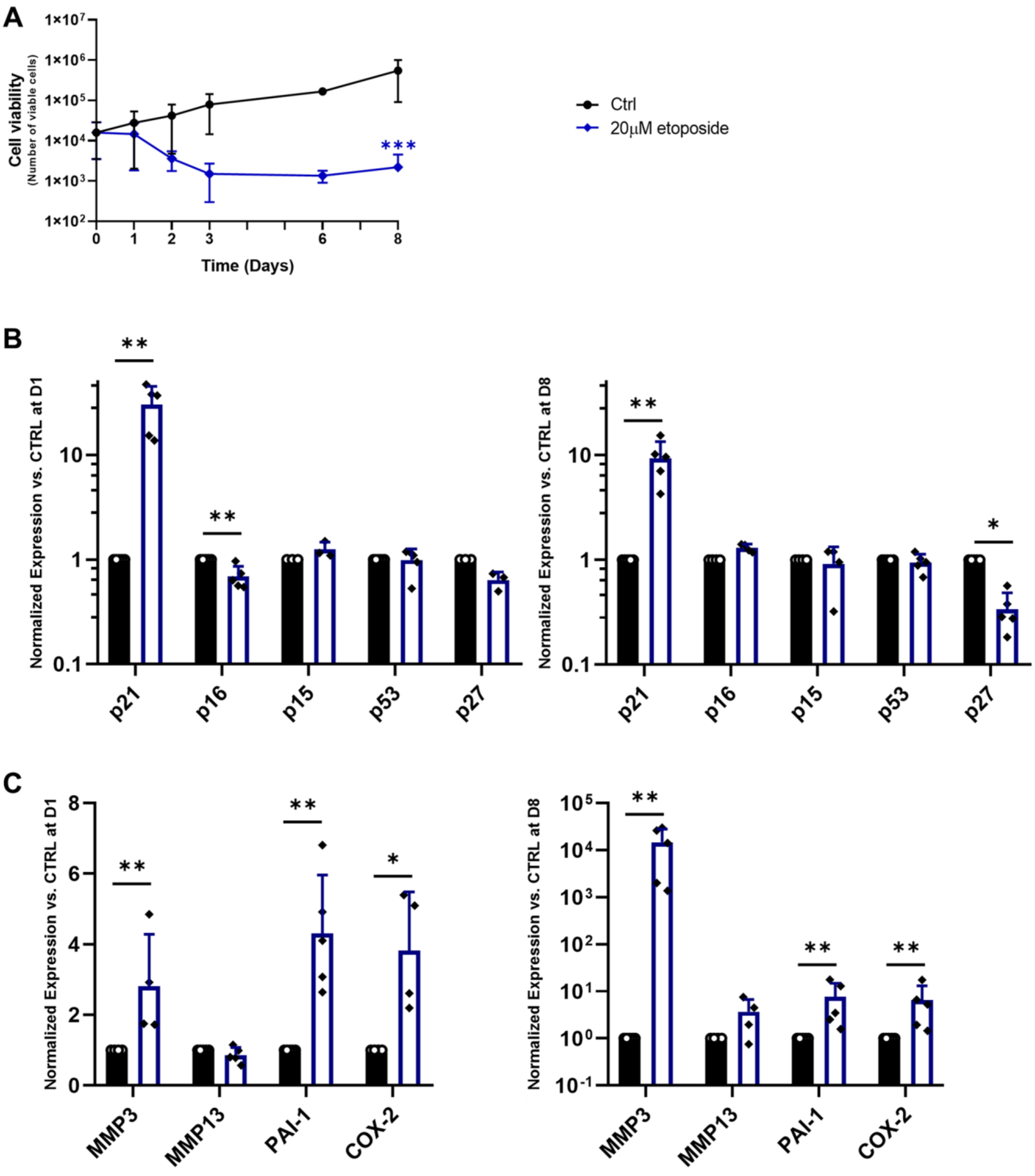

Supplementary Figure 1. Impact of Etoposide on the TC28a2 chondrocyte cell line. TC28a2 were treated with etoposide (blue) at 20 µM for 24 h and then cultured in normal media for the length of the experiment. (**A**) The number of viable cells was assessed by trypan blue exclusion dye. Data are shown as mean ± SD, (n = 3). P-values were calculated by the two-way ANOVA test. $^{***}p$ < 0,001. (**B**, **C**) The expressions of cyclin-dependent kinase inhibitors (**B**) and SASP markers (**C**) were evaluated by RT-qPCR at the indicated times. Data are shown as mean ± SD, ($n \geq 3$). P-values were calculated by Mann-Whitney test, $^{*}p \leq 0.05$; $^{**}p < 0.01$; $^{***}p < 0.001$.

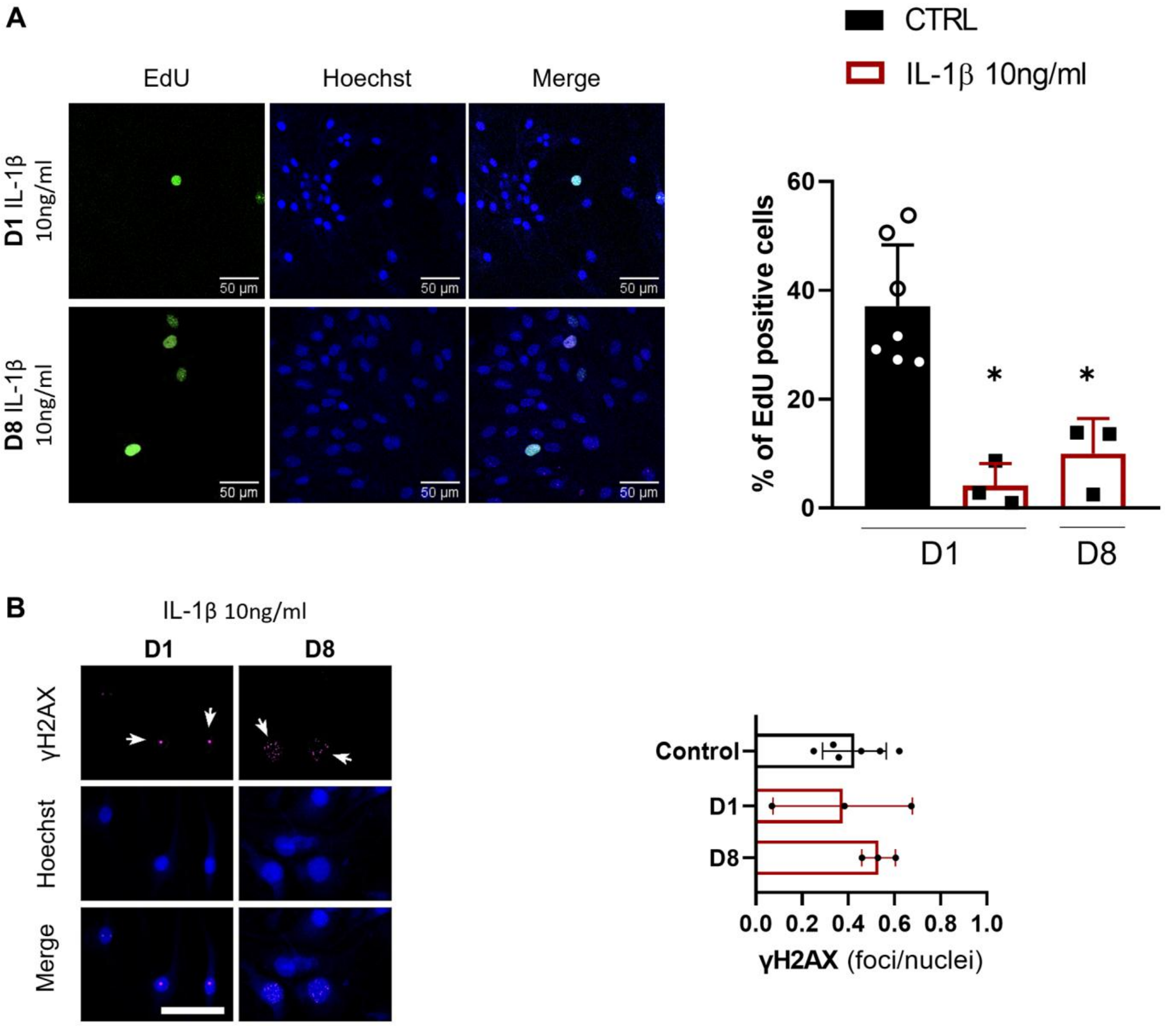

Supplementary Figure 2. Impact of high-dose IL-1β-treatment on HACs proliferation and DNA damage. HACs were treated with IL-1β at 10 ng/mL for the length of the experiment, the results are compared with the 1 ng/mL IL1β treatment presented in Figures 2 and 4. (**A**) EdU was used to identify proliferative cells and Hoechst staining to visualize the nucleus at day 1 and 8. The images were analyzed by quantification of positive cells for EdU normalized versus the total number of cells obtained with the Hoechst staining at each time. (**B**) γH2AX immunofluorescence was used to identify DNA damage-associated foci and Hoechst staining to visualize the nucleus at day 1 and day 8. Quantification of the average number of foci per nuclei is shown. Scale bars = 50 µm. Data are shown as mean ± SD, (*n* = 3). *P*-values were calculated by Kruskal-Wallis test, $^{*}p \leq 0.05$; $^{***}p < 0.001$.
